# Impact of myocardial bridge on lesion morphology and clinical outcomes in patients undergoing IVUS-guided PCI for LAD CTO

**DOI:** 10.3389/fcvm.2025.1648233

**Published:** 2025-07-21

**Authors:** Xi Wu, Mingxing Wu, Haobo Huang, Zhe Liu, He Huang, Lei Wang

**Affiliations:** Department of Cardiology, Xiangtan Central Hospital (The Affiliated Hospital of Hunan University), Xiangtan, Hunan, China

**Keywords:** myocardial bridge, chronic total occlusion, left anterior descending artery, percutaneous coronary intervention, intravascular ultrasound

## Abstract

**Introduction:**

Myocardial bridge (MB) is increasingly recognized for its potential role in coronary artery disease. However, its impact on lesion morphology and clinical outcomes in patients with left anterior descending (LAD) chronic total occlusion (CTO) undergoing intravascular ultrasound (IVUS)-guided percutaneous coronary intervention (PCI) remains unclear.

**Methods:**

This single-center retrospective study analyzed 256 patients who underwent IVUS-guided PCI for LAD CTO between 2016 and 2022. Patients were divided into MB (*n* = 61) and non-MB (*n* = 195) groups based on IVUS findings. Lesion characteristics, stent strategy, and 2-year clinical outcomes were compared.

**Results:**

MB was identified in 23.8% of patients. Compared with the non-MB group, MB patients had significantly shorter CTO length (17.71 mm vs. 21.31 mm, *P* < 0.001), less calcification (29.5% vs. 47.7%, *P* = 0.018), and more proximal lesion distribution (41.0% vs. 20.0%, *P* = 0.001). Despite these favorable anatomical features, the MB group had higher rates of major adverse cardiovascular events (MACE) (19.7% vs. 8.7%, *P* = 0.033) and clinically driven target lesion revascularization (18.0% vs. 6.7%, *P* = 0.016). MB was an independent predictor of MACE (HR = 2.173, P = 0.021).

**Discussion:**

MB is associated with distinct morphological features and worse clinical outcomes in LAD CTO patients undergoing PCI. Its presence may require careful procedural planning and personalized revascularization strategies to reduce long-term risks.

## 1 Introduction

Coronary arteries conventionally traverse the epicardial surface of the heart, embedded within subepicardial connective tissue. However, in certain individuals, a segment of a coronary artery courses intramyocardially and becomes surrounded by myocardial fibers—a configuration identified as a myocardial bridge (MB), with the embedded portion referred to as a “tunneled artery” (1). The left anterior descending artery (LAD), especially its mid-to-distal segments, is most commonly involved, with MB reported in as many as 67%–98% of cases (2).

Historically regarded as a benign anatomical variant, MBs have reemerged as clinically significant due to accumulating evidence linking them to unfavorable cardiovascular outcomes. These include myocardial ischemia, acute coronary syndromes (ACS), coronary vasospasm, arrhythmogenic events, and even sudden cardiac death (3, 4). The proposed pathophysiological mechanism involves dynamic compression of the bridged segment during systole, which may alter coronary flow dynamics and impair endothelial function in adjacent arterial regions (1). Interestingly, MBs exhibit a paradoxical role in the context of atherosclerosis: while the tunneled segment tends to be protected from plaque formation due to mechanical shielding, the arterial region proximal to the MB frequently demonstrates enhanced plaque burden and vulnerability. This is likely attributable to altered shear stress and flow turbulence (5). This duality—simultaneously protective and predisposing—has led to MBs being described as a “double-edged sword” in coronary artery disease (6).

The observed prevalence of MBs varies substantially across different diagnostic modalities. Postmortem examinations have reported prevalence rates approaching 86% (1), whereas imaging techniques such as intravascular ultrasound (IVUS) and computed tomography have identified MBs in approximately 22%–40% of individuals (7). Conversely, coronary angiography is less sensitive in detecting MBs, with detection rates often below 5% (2). Recent retrospective analyses have highlighted the potential relevance of MBs in patients presenting with chronic total occlusion (CTO) of the LAD. Preliminary data indicate that MBs may be more prevalent in LAD CTO lesions compared to non-occlusive counterparts, with implications for procedural planning and long-term outcomes after percutaneous coronary intervention (PCI) (8). Importantly, stenting within MB-involved segments has been associated with increased risks of target lesion failure (TLF), possibly due to factors such as mechanical compliance mismatch, elastic recoil, or inadequate stent deployment (8). Despite these findings, direct comparative investigations of LAD CTO lesions with and without MBs are sparse. It remains unclear whether significant anatomical or procedural differences exist between these two groups, or how the presence of MBs influences intravascular imaging interpretation, stent strategy, and clinical outcomes following PCI. This study, therefore, seeks to conduct a comparative evaluation of LAD CTO lesions with and without MB, emphasizing distinctions in lesion architecture, stent deployment characteristics, and post-intervention outcomes. Through this analysis, we aim to enhance procedural planning and advance understanding of the complex interaction between MBs and severe coronary artery occlusions.

## 2 Materials and methods

### 2.1 Study participants

**Figure 1:**
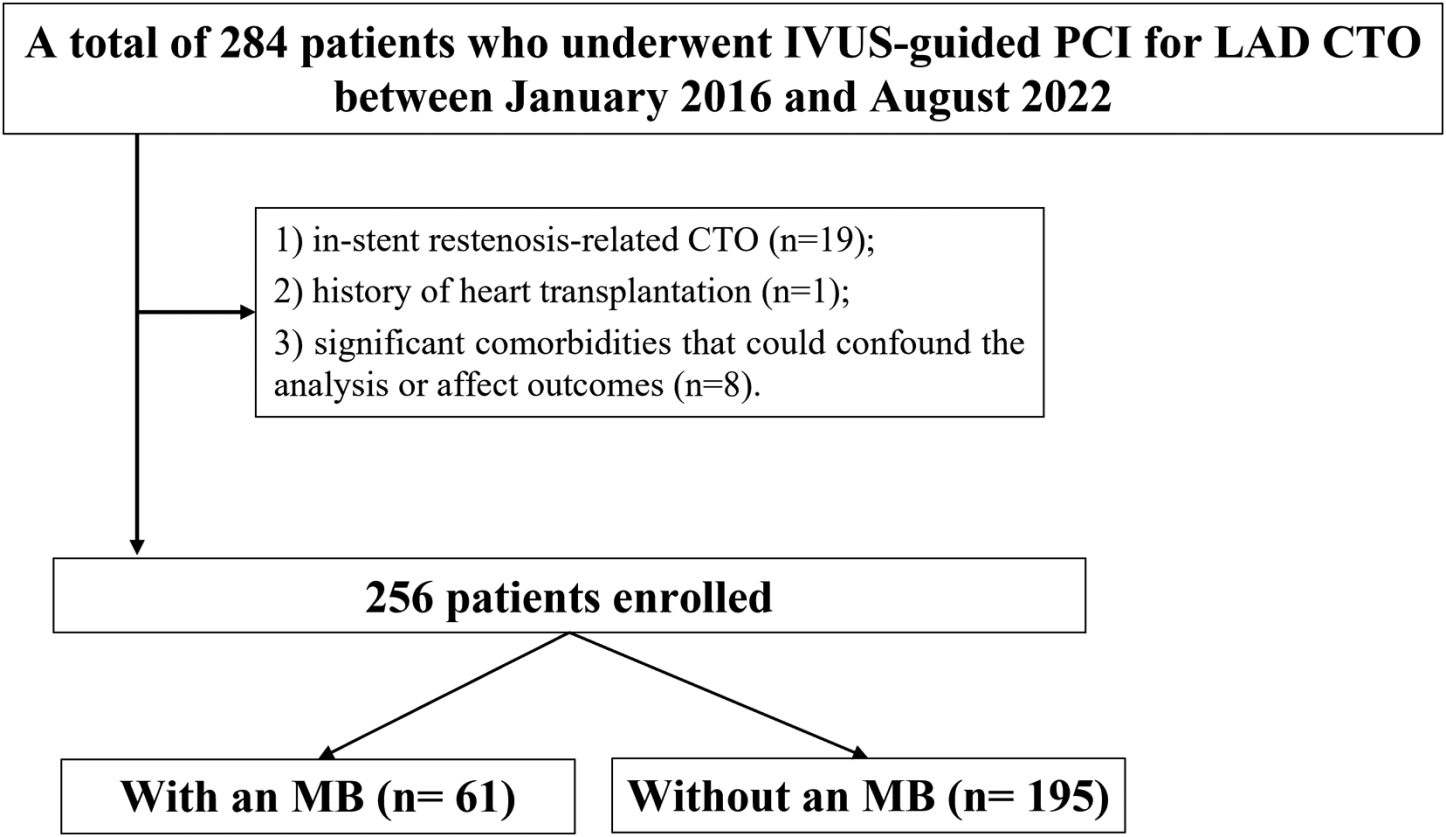
Study flowchart. MB, myocardial bridge; CTO, chronic total occlusion; LAD, left anterior descending; IVUS, intravascular ultrasound; PCI, percutaneous coronary intervention.

This was a single-center, retrospective observational study conducted at the Department of Cardiology, Xiangtan Central Hospital. A total of 256 consecutive patients who underwent IVUS-guided successful PCI for CTO lesions in the LAD artery between January 2016 and August 2022 were included. For patients without documented clinical evidence of occlusion duration, the chronicity of the lesion was assessed based on angiographic features indicative of long-standing occlusion (9). To ensure adequate assessment of MB, only patients with >40 mm analyzable IVUS image length distal to the LAD ostium were included (10). Inclusion criteria: successful PCI for *de novo* LAD CTO lesions; use of IVUS during the procedure; analyzable IVUS pullbacks with adequate segment length for MB detection. Patients were excluded if they had in-stent restenosis-related CTO, a history of heart transplantation, or significant comorbidities that could confound the analysis or affect outcomes. These included severe hepatic or renal dysfunction, hyperthyroidism, active malignancy with extensive metastatic spread, and bleeding disorders (Figure 1). All patients presented with symptoms consistent with myocardial ischemia, predominantly effort-induced angina pectoris or angina-equivalent symptoms such as dyspnea on exertion. Objective evidence of ischemia was evaluated prior to PCI decision-making based on a combination of clinical presentation, resting or stress electrocardiography, and echocardiographic assessment of regional wall motion abnormalities when clinically indicated. In addition, coronary angiography demonstrated LAD chronic total occlusion with impaired distal perfusion, supporting the diagnosis of significant myocardial ischemia. Demographic characteristics, clinical comorbidities, laboratory parameters, angiographic and IVUS imaging data, procedural details, and long-term clinical outcomes were retrospectively collected from the hospital's electronic medical records and imaging archives. The study complied with the ethical principles outlined in the Declaration of Helsinki (2013 revision) and was approved by the Ethics Committee of Xiangtan Central Hospital (Approval No. X201853). Written informed consent was obtained from all participants. For certain cases where written consent was not feasible, verbal consent was documented in accordance with institutional policy.

### 2.2 CTO procedures

All CTO procedures were conducted by experienced interventionalists, with procedural strategies determined individually at the operator's discretion. Upon successful guidewire crossing, balloon predilation was initially performed under angiographic guidance. IVUS imaging was then utilized to verify the intraluminal position of the guidewire distally, evaluate the lesion architecture, and delineate optimal stent landing zones. Lesion preparation was undertaken when indicated. Stents were deployed in reference vessel segments demonstrating a plaque burden of less than 50%, as assessed by IVUS. In cases where an MB was located distal to the lesion, stent placement into the MB segment was generally avoided. Exceptions were made in the presence of significant dissection involving the MB or when critical disease existed just proximal to the MB, necessitating extension of the stent into the bridged segment. Technical success was defined by restoration of antegrade TIMI grade 3 flow and achieving residual diameter stenosis <30% in the treated segment. Total procedure time was recorded from the initiation of vascular access to the withdrawal of the final catheter. In this study cohort, all lesions were treated with second-generation DES implantation, and neither bioresorbable scaffolds nor drug-coated balloon strategies were utilized.

### 2.3 Periprocedural pharmacotherapy

All patients received dual antiplatelet therapy (DAPT) consisting of aspirin (100 mg/day) and clopidogrel (75 mg/day). For patients who had not been on chronic DAPT for at least 7 days prior to the procedure, a loading dose was administered 24 h before the intervention, consisting of aspirin (300 mg) and either clopidogrel (300 mg) or ticagrelor (180 mg), in accordance with current guideline recommendations. Post-procedurally, patients continued DAPT with aspirin (100 mg/ day) and clopidogrel (75 mg/day) for 12 months. Additional pharmacotherapy—including statins, beta-blockers, angiotensin-converting enzyme inhibitors (ACEIs) or angiotensin receptor blockers (ARBs), and nitrates—was prescribed as clinically indicated.

### 2.4 Angiographic analysis strategy and assessment

Coronary angiographic evaluation was conducted by an experienced interpreter (H.H.) who was blinded to all clinical and IVUS data. Quantitative analysis was performed using the QAngioXA software (Medis Medical Imaging Systems, Leiden, the Netherlands). The length of the CTO lesion was determined based on contrast opacification of the distal vessel using either antegrade or retrograde approaches, including simultaneous bilateral injections when appropriate. Lesion complexity was assessed utilizing the J-CTO score, as defined by the Multicenter CTO Registry of Japan (11). The extent of collateral circulation was graded according to the Rentrop classification system (12).

### 2.5 IVUS imaging and analysis

Following successful guidewire advancement, all CTO lesions underwent balloon predilation prior to prestenting IVUS imaging. To reduce the risk of vasospasm, an intracoronary dose of 100–200 μg nitroglycerin was routinely administered before image acquisition. Two IVUS catheters were used during the study period: the 40 MHz Atlantis SR catheter (Boston Scientific, USA) from 2016 to 2019, and the OptiCross catheter (Boston Scientific, USA) from 2019 to 2022. Both devices were compatible with the iLab IVUS system and provided similar image quality and acquisition characteristics. The IVUS catheter was positioned distally beyond the lesion and withdrawn proximally toward the aorta under fluoroscopy at a consistent speed of 0.5 mm/s or 1.0 mm/s. Imaging sequences were digitally stored. Offline interpretation was performed using QIvus® software (Medis, Leiden, the Netherlands), a dedicated IVUS analysis platform. Quantitative and qualitative measurements were independently performed by two trained reviewers blinded to clinical data. In the event of disagreement, a third senior analyst adjudicated the final value. Identical analysis protocols and scoring criteria were employed for both catheter systems to maintain methodological uniformity.

**Figure 2:**
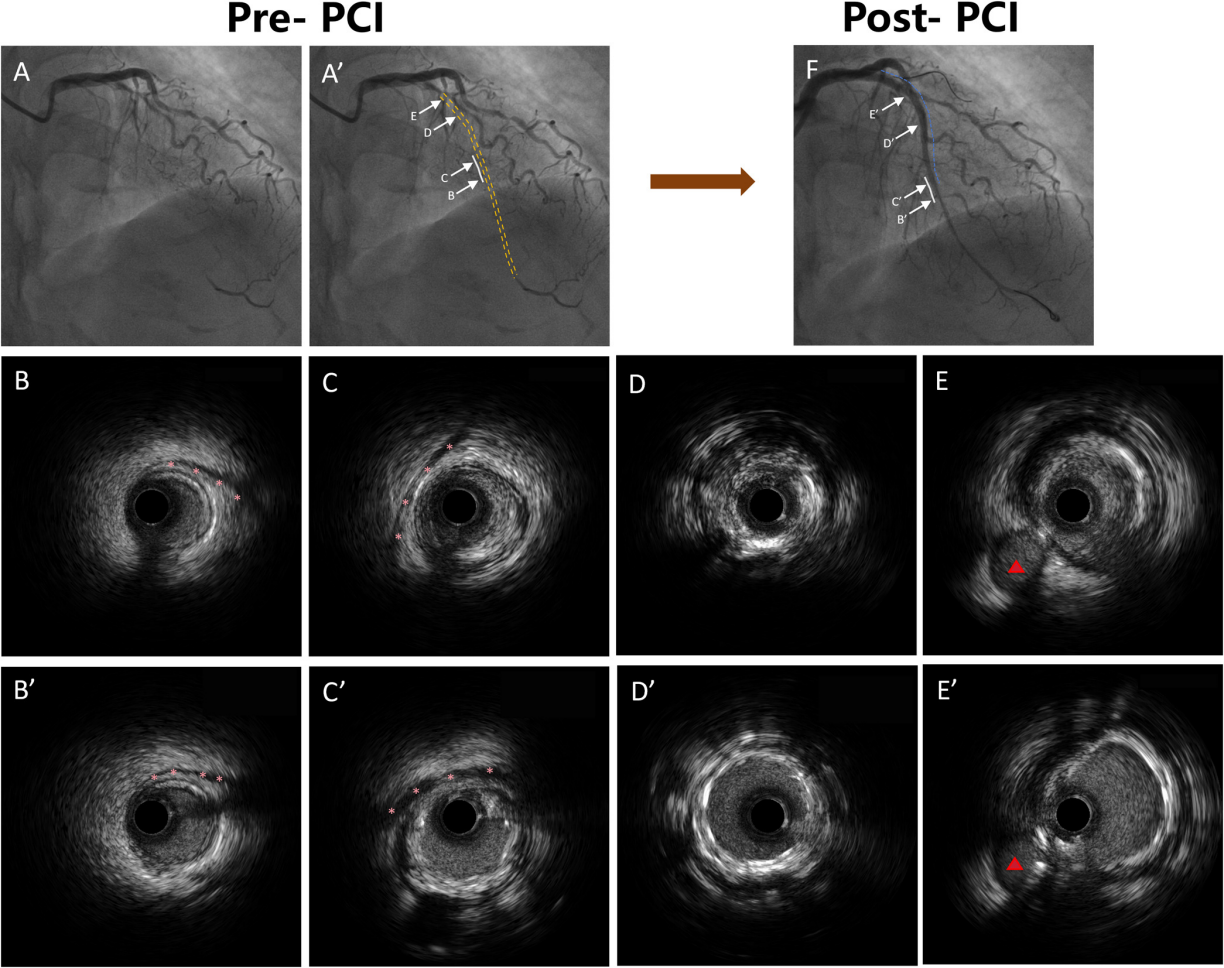
Representative coronary angiography and IVUS images demonstrating MB morphology and stent implantation strategy. **(A)** A’: Coronary angiography demonstrating the LAD with MB segments marked. The white solid lines indicate MB segments, and the yellow dashed line indicates the occluded segment identified by angiography. **(B)** B’: IVUS image showing the distal segment of the MB without any plaque formation. The pink asterisks indicate the stent edges. **(C)** C’: IVUS image showing plaque formation in the proximal segment of the MB, with the stent extending into the bridged segment. The pink asterisks indicate the stent edges. **(D)** D’: IVUS image showing stent implantation within the CTO segment without MB. **(E)** E’: IVUS image showing stent implantation in the proximal LAD segment outside of the CTO lesion. The red triangles indicate the diagonal branch. **(F)** Coronary angiography image showing the LAD after stent implantation. The blue dashed lines indicate the stented segments, and the white solid lines indicate the MB segments. IVUS, intravascular ultrasound; MB, myocardial bridge; LAD, left anterior descending; PCI, percutaneous coronary intervention.

All analyses were conducted according to contemporary recommendations for IVUS imaging acquisition, interpretation, and reporting, as outlined in expert consensus guidelines (13) on intravascular imaging and physiologic assessment. An MB was identified as a segment of epicardial coronary artery showing systolic compression surrounded by echolucent muscular tissue on IVUS (14) (Figure 2). Within this segment, the following parameters were evaluated: minimum lumen area (MLA), plaque burden at the MLA, maximum MB thickness, total MB length, and diastolic vessel restriction, calculated as (1—diastolic vessel area/interpolated reference vessel area) (14). True CTO length was determined by coregistering IVUS images with angiography via fiduciary landmarks. The IVUS-defined CTO segment was characterized by cross-sections lacking a smooth, concave lumen contour, distinguishing it from adjacent reference regions. CTO length was then calculated based on the pullback speed and duration. For manually withdrawn catheters, measurements were derived from the coregistered angiogram. Extraplaque tracking was defined as guidewire passage outside the plaque but within the adventitia, recognized by the absence of the classic three-layer vessel wall pattern (15). Stent expansion was defined as the ratio of minimum stent area (MSA) to the average lumen area of the proximal and distal reference segments. All IVUS measurements were obtained during phases of maximal vessel diameter, presumed to correspond with diastole. Anatomical assessments from both IVUS and angiographic data were independently reviewed by two experienced interventional cardiologists (X.W. and L.W.) blinded to patient presentation and laboratory data. Inter-observer and intra-observer agreement were excellent, with *κ* coefficients of 0.90 and 0.93, respectively.

### 2.6 Follow-up and clinical outcomes

Patients were followed up at 1, 6, and 12 months post-discharge and annually thereafter. Follow-up data were collected via outpatient visits, hospital records, telephone interviews, and verification through referring physicians or national mortality databases. The primary outcome was the occurrence of major adverse cardiovascular events (MACE), defined as a composite of cardiac death, target vessel myocardial infarction (MI), clinically driven target lesion revascularization (TLR), or in-stent thrombosis (IST), as per the Academic Research Consortium definitions (16). Clinically driven TLR was defined as revascularization performed in the presence of recurrent ischemic symptoms or objective evidence of myocardial ischemia, in combination with angiographic restenosis of ≥50% within the target lesion, in accordance with guideline recommendations.

### 2.7 Statistical analysis

All statistical analyses were conducted using IBM SPSS Statistics version 26.0 (IBM Corp., Armonk, NY, USA). The Kolmogorov–Smirnov test was employed to assess the distributional normality of continuous variables. Variables following a normal distribution were presented as mean ± standard deviation (SD) and compared between groups using independent-samples t-tests. For variables not normally distributed, data were expressed as median and interquartile range [M (P25, P75)] and compared using the Mann–Whitney *U* test. Categorical variables were summarized as frequencies and percentages [*n* (%)] and analyzed using either Pearson's *χ*^2^ test or Fisher's exact test, depending on cell counts. All statistical evaluations were two-sided, and a *p*-value < 0.05 was considered to indicate statistical significance. Variables identified as potential predictors of MACE in univariate analysis were subsequently entered into a multivariate Cox proportional hazards regression model using backward stepwise selection to determine independent predictors of adverse outcomes. The cumulative incidence of MACE was estimated using Kaplan–Meier survival curves, and differences between groups were assessed with the log-rank test. A two-tailed *P* value < 0.05 was defined as the threshold for statistical significance.

## 3 Results

A total of 256 patients with LAD CTO were included, of whom 61 (23.8%) had coexisting MB. As shown in Table 1, baseline demographic characteristics, cardiovascular risk factors, and laboratory indices were generally balanced between the MB and non-MB groups, with no statistically significant differences observed.

**Table 1:** Baseline characteristics.

Variable	All (*n* = 256)	With an MB (*n* = 61)	Without an MB (*n* = 195)	*P* value
Age, years	65.00 (61.00, 71.00)	63.00 (59.00, 70.00)	67.00 (64.00, 71.00)	0.123
Male, *n* %	158 (61.7%)	37 (60.7%)	121 (62.1%)	0.842
Prior hypertension, *n* %	128 (50.0%)	30 (49.2%)	98 (50.3%)	0.887
Prior hyperlipidemia, *n* %	99 (38.7%)	21 (34.4%)	78 (40.0%)	0.504
Prior diabetes mellitus, *n* %	70 (27.3%)	20 (32.8%)	50 (25.6%)	0.293
Prior stroke, *n* %	14 (5.5%)	4 (6.6%)	10 (5.1%)	0.915
Smoking, *n* %	124 (48.4%)	31 (50.8%)	93 (47.7%)	0.779
Chronic kidney diseasea^a^, *n* %	9 (3.5%)	5 (8.2%)	4 (2.1%)	0.060
Peripheral artery disease, *n* %	34 (13.3%)	9 (14.8%)	25 (12.8%)	0.863
Prior myocardial infarction, *n* %	77 (30.1%)	16 (26.2%)	61 (31.3%)	0.554
Prior PCI, *n* %	27 (10.5%)	7 (11.5%)	20 (10.3%)	0.974
Laboratory biomarkers				
Platelet count, 10^9 ^/L	251.12 (235.24, 272.83)	249.75 (234.98, 268.73)	252.98 (236.02, 274.49)	0.445
TG, mmol/L	1.93 (1.69, 2.17)	1.94 (1.75, 2.16)	1.93 (1.69, 2.17)	0.709
TC, mmol/L	5.36 (5.04, 5.66)	5.34 (5.04, 5.78)	5.38 (5.05, 5.66)	0.886
HDL, mmol/L	1.24 (1.16, 1.35)	1.22 (1.16, 1.37)	1.25 (1.16, 1.35)	0.728
LDL, mmol/L	3.32 (3.09, 3.63)	3.33 (3.11, 3.60)	3.32 (3.09, 3.63)	0.766
Lp(a), mg/L	203.21 (168.46, 250.59)	201.62 (171.11, 254.71)	204.15 (167.33, 248.94)	0.697
AST, U/L	112.71 (89.65, 133.21)	120.45 (97.93, 138.11)	110.45 (88.57, 130.92)	0.051
ALT, U/L	49.35 (38.34, 63.60)	49.37 (41.34, 64.36)	49.32 (37.39, 63.05)	0.467
TBIL, μmol/L	16.34 (15.13, 18.00)	16.37 (15.14, 18.33)	16.28 (15.13, 17.96)	0.634
Uric acid, μmol/L	482.89 (433.10, 541.59)	466.49 (414.73, 514.59)	489.27 (441.03, 550.92)	0.070
Scr, μmol/L	88.92 (84.51, 95.77)	88.46 (84.35, 94.20)	89.21 (84.65, 96.04)	0.731
eGFR, ml/min per 1.732 m^2^	99.07 (91.64, 107.72)	102.92 (90.93, 114.04)	98.28 (91.77, 107.03)	0.476
Pharmacologic therapy				
DAPT, *n* %	256 (100.0%)	61 (100.0%)	195 (100.0%)	1.000
Statins, *n* %	237 (92.6%)	59 (96.7%)	178 (91.3%)	0.256
ACEI or ARB, *n* %	190 (74.2%)	42 (68.9%)	148 (75.9%)	0.352
Beta-blockers, *n* %	150 (58.6%)	38 (62.3%)	112 (57.4%)	0.601
Aldosterone antagonists, *n* %	48 (18.8%)	12 (19.7%)	36 (18.5%)	0.981
Nitrates, *n* %	67 (26.2%)	15 (24.6%)	52 (26.7%)	0.876
Calcium channel blockers, *n* %	36 (14.1%)	7 (11.5%)	29 (14.9%)	0.649

Continuous variables were expressed as median (interquartile range). Categorical variables were expressed as number (percentage).

MB, myocardial bridge; PCI, percutaneous coronary intervention; DAPT, dual antiplatelet therapy; ACEI, angiotensin-converting enzyme inhibitor; ARB, angiotensin-receptor blocker; TG, triglycerides; TC, total cholesterol; HDL, high-density lipoprotein; LDL, low-density lipoprotein; Lp(a), lipoprotein(a); AST, aspartate aminotransferase; ALT, alanine aminotransferase; TBIL, total bilirubin; Scr, serum creatinine; eGFR, estimated glomerular filtration rate.

^a^ Estimated glomerular filtration rate <60 ml/min/1.73 m^2^ using the Modification of Diet in Renal Disease study equation.

Angiographic and procedural characteristics are summarized in Table 2. The MB group exhibited a significantly shorter lesion length (19.36 ± 2.31 mm vs. 20.37 ± 2.58 mm, *P* = 0.004), more frequent ostial-proximal location (41.0% vs. 20.0%, *P* = 0.001), and shorter calcium arc length (11.55 ± 0.41 mm vs. 12.39 ± 0.52 mm, *P* < 0.001). The total stent length was also significantly reduced in MB patients (69.17 ± 2.93 mm vs. 71.46 ± 4.13 mm, *P* < 0.001). Other parameters including device diameter, retrograde approach rate, and procedural time were comparable between groups.

**Table 2:** Angiographic and procedural findings.

Variable	All (*n* = 256)	With an MB (*n* = 61)	Without an MB (*n* = 195)	*P* value
Multivessel disease^a^, *n* %	88 (34.4%)	19 (31.1%)	69 (35.4%)	0.650
Reattempt CTO PCI, *n* %	15 (5.9%)	4 (6.6%)	11 (5.6%)	1.000
CTO length, mm	20.13 ± 2.55	19.36 ± 2.31	20.37 ± 2.58	0.004
CTO length >20 mm, *n* %	111 (43.4%)	24 (39.3%)	87 (44.6%)	0.563
Ostial to proximal lesion location, *n* %	64 (25.0%)	25 (41.0%)	39 (20.0%)	0.001
Lesion length, mm	43.35 ± 3.38	42.60 ± 3.77	43.58 ± 3.22	0.069
Calcification, *n* %	71 (27.7%)	13 (21.3%)	58 (29.7%)	0.262
Calcium length, mm	12.19 ± 0.61	11.55 ± 0.41	12.39 ± 0.52	<0.001
Abrupt proximal cap, *n* %	129 (50.4%)	29 (47.5%)	100 (51.3%)	0.716
Lesion bend, *n* %	29 (11.3%)	6 (9.8%)	23 (11.8%)	0.849
J-CTO score ≥2, *n* %	114 (44.5%)	26 (42.6%)	88 (45.1%)	0.844
Rentrop classification grade 3, *n* %	128 (50.0%)	33 (54.1%)	95 (48.7%)	0.557
Post-PCI in-segment^b^				
Reference vessel diameter, mm	3.19 ± 0.47	3.16 ± 0.41	3.20 ± 0.48	0.498
Minimum lumen diameter, mm	2.52 ± 0.20	2.51 ± 0.11	2.52 ± 0.22	0.853
Diameter stenosis, %	23.17 ± 4.97	23.22 ± 5.38	23.15 ± 4.85	0.929
Post-PCI distal vessel				
Reference vessel diameter, mm	1.58 ± 1.29	1.39 ± 1.10	1.64 ± 1.34	0.143
Minimum lumen diameter, mm	1.13 ± 0.60	1.11 ± 0.45	1.14 ± 0.63	0.678
Diameter stenosis, %	30.05 ± 4.59	30.12 ± 4.12	30.03 ± 4.74	0.887
Procedural findings				
Final guidewire crossing technique				0.268
Antegrade guidewire escalation, *n* %	130 (50.8%)	28 (45.9%)	102 (52.3%)	
Antegrade dissection reentry, *n* %	25 (9.8%)	7 (11.5%)	18 (9.2%)	
Retrograde guide wire escalation, *n* %	26 (10.2%)	10 (16.4%)	16 (8.2%)	
Retrograde dissection reentry, *n* %	75 (29.3%)	16 (26.2%)	59 (30.3%)	
Retrograde attempt, *n* %	88 (34.4%)	19 (31.1%)	69 (35.4%)	0.650
Total stent length, mm	70.92 ± 3.99	69.17 ± 2.93	71.46 ± 4.13	<0.001
Maximum device diameter, mm	3.30 ± 2.58	3.41 ± 2.62	3.27 ± 2.57	0.708
Maximum balloon inflation pressure, atm	18.43 ± 2.91	18.59 ± 3.26	18.37 ± 2.80	0.632
Procedure time, min	50.39 ± 5.87	48.84 ± 7.83	50.87 ± 5.03	0.061
Radiation exposure dose, Gy	2.1 (1.5, 2.7)	2.1 (1.6, 2.6)	2.1 (1.4, 2.8)	0.944
Contrast media volume, ml	272.94 ± 13.57	270.51 ± 12.03	273.70 ± 13.95	0.085

Continuous variables were expressed as mean ± SD, or median (interquartile range). Categorical variables were expressed as number (percentage).

MB, myocardial bridge; PCI, percutaneous coronary intervention; CTO, chronic total occlusion.

^a^ Defined as the presence of >50% diameter stenosis in 2 or more major epicardial arteries.

^b^ In-segment includes stent and 5 mm proximal and distal reference from each stent edge.

IVUS analysis (Table 3) revealed that MB patients had significantly shorter CTO lesion lengths (17.71 ± 3.21 mm vs. 21.31 ± 2.44 mm, *P* < 0.001) and a lower prevalence of calcification within the CTO segment (29.5% vs. 47.7%, *P* = 0.018). No significant intergroup differences were observed in minimum stent area, stent expansion percentage, or other IVUS-derived procedural outcomes.

**Table 3:** Intravascular ultrasound findings.

Variable	All (*n* = 256)	With an MB (*n* = 61)	Without an MB (*n* = 195)	*P* value
CTO length, mm	20.45 ± 3.05	17.71 ± 3.21	21.31 ± 2.44	<0.001
CTO length >20 mm, *n* %	106 (41.4%)	18 (29.5%)	88 (45.1%)	0.044
Extraplaque tracking, *n* %	59 (23.0%)	15 (24.6%)	44 (22.6%)	0.877
Extraplaque length, mm	31.70 ± 3.53	31.78 ± 3.05	31.67 ± 3.67	0.816
Lesion length, mm	49.67 ± 5.53	48.77 ± 5.62	49.95 ± 5.48	0.150
Maximum plaque burden, %	84.26 ± 5.48	84.56 ± 5.03	84.16 ± 5.62	0.602
Calcification in CTO lesion, *n* %	111 (43.4%)	18 (29.5%)	93 (47.7%)	0.018
Maximum arc of calcium,°	125.34 ± 21.30	125.90 ± 22.77	125.16 ± 20.88	0.822
Dissection, *n* %	102 (39.8%)	24 (39.3%)	78 (40.0%)	1.000
Dissection extended into an MB, *n* %	–	6 (9.8)	–	–
Reference minimum lumen area, mm^2^	3.52 ± 1.33	3.56 ± 1.33	3.51 ± 1.33	0.799
Reference maximum plaque burden, %	58.32 ± 3.41	58.74 ± 3.22	58.19 ± 3.47	0.262
MB segment				
Distance from LAD ostium to MB, mm	–	38.52 ± 6.43	–	–
Total MB length, mm	–	9.53 ± 2.52	–	–
Maximum thickness of MB, mm	–	0.49 ± 0.09	–	–
Diastolic vessel area at max compression site, mm^2^	–	4.41 ± 0.53	–	–
Diastolic vessel restriction, %	–	19.47 ± 4.43	–	–
Minimum lumen area, mm^2^	–	2.39 ± 0.64	–	–
Plaque burden at minimum lumen area site, %	–	41.02 ± 4.32	–	–
Postprocedure findings				
MSA, mm^2^	5.18 ± 3.11	5.09 ± 2.91	5.21 ± 3.17	0.780
Stent expansion, %	70.51 ± 3.82	70.70 ± 2.97	70.45 ± 4.06	0.606
Rate of MSA in the MB, when stented, *n* %	–	31 (50.8)	–	–

Continuous variables were expressed as mean ± SD. Categorical variables were expressed as number (percentage).

MB, myocardial bridge; CTO, chronic total occlusion; MSA, minimum stent area; LAD, left anterior descending artery.

During the 2-year follow-up (Table 4), the incidence of MACE was significantly higher in the MB group compared to the control group (19.7% vs. 8.7%, *P* = 0.033). Similarly, the rate of clinically driven TLR was increased in patients with MB (18.0% vs. 6.7%, *P* = 0.016). No significant differences were found in cardiac death, target vessel myocardial infarction (TVMI), or in-stent thrombosis.

**Table 4:** 2-year clinical outcomes.

Variable	All (*n* = 256)	With an MB (*n* = 61)	Without an MB (*n* = 195)	*P* value
MACE, *n* %	29 (11.3%)	12 (19.7%)	17 (8.7%)	0.033
Cardiac death, *n* %	1 (0.4%)	1 (1.6%)	0 (0.0%)	0.538
Target vessel MI, *n* %	5 (2.0%)	1 (1.6%)	4 (2.1%)	1.000
Clinically driven TLR, *n* %	24 (9.4%)	11 (18.0%)	13 (6.7%)	0.016
In-stent thrombosis, *n* %	3 (1.2%)	2 (3.3%)	1 (0.5%)	0.284

Categorical variables were expressed as number (percentage).

MB, myocardial bridge; MACE, major adverse cardiovascular events; MI, myocardial infarction; TLR, target lesion revascularization.

Univariate logistic regression analysis (Table 5) demonstrated that MB was significantly associated with MACE. In the multivariate model, after adjustment for age, sex, hypertension, dyslipidemia, diabetes mellitus, and chronic kidney disease, MB remained an independent predictor of 2-year MACE (HR: 2.173, 95% CI: 1.031–4.667, *P* = 0.021).

**Table 5:** Univariate and multivariate Cox regression analyses showing independent predictors of MACE in patients with LAD CTO.

Variables	Univariate analysis			Multivariate analysis		
	HR	95% CI	*P* value	HR	95% CI	*P* value
Age	1.847	1.155–2.953	0.018	1.122	0.623–2.012	0.704
Hypertension	1.008	0.561–1.811	0.977			
Diabetes mellitus	1.251	0.695–2.246	0.455			
MB	2.072	1.168–3.674	0.012	2.173	1.031–4.667	0.021
Prior MI	1.065	0.588–1.929	0.833			
LDL	1.125	0.626–2.022	0.693			

MB, myocardial bridge; MACE, major adverse cardiovascular events; HR, hazard ratios; CI, confidence interval; CTO, chronic total occlusion; LAD, left anterior descending artery; MI, myocardial infarction.

**Figure 3:**
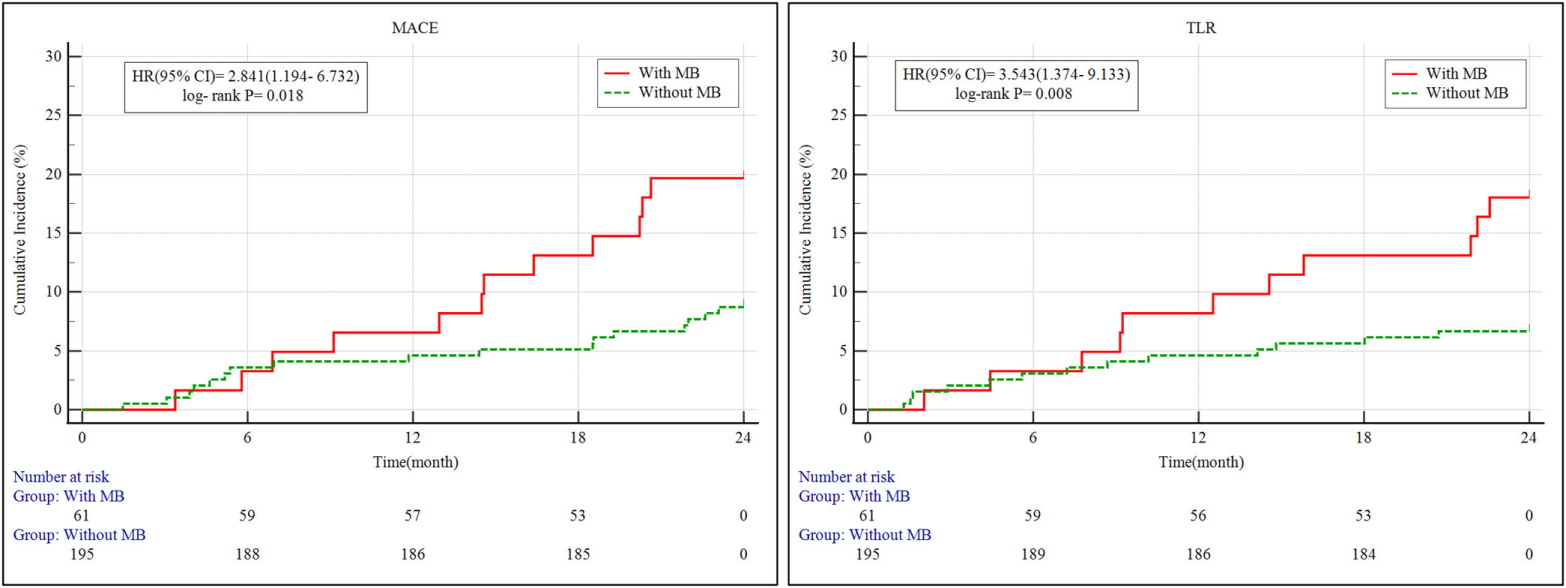
Kaplan–Meier survival curves of MACE and clinically driven TLR for 2 years. MB, myocardial bridge; MACE, major adverse cardiovascular events; TLR, target lesion revascularization; 95% CI, 95% confidence intervals; HR, hazard ratio.

Kaplan–Meier survival analysis revealed that patients with MB exhibited a significantly higher cumulative incidence of both MACE and clinically driven TLR over the 2-year follow-up period compared to those without MB (Figure 3). Specifically, the incidence of MACE was significantly elevated in the MB group (log-rank *P* = 0.018), with a hazard ratio (HR) of 2.841 [95% confidence interval (CI): 1.194–6.732]. Likewise, the risk of clinically driven TLR was significantly higher in the MB group, with an HR of 3.543 (95% CI: 1.374–9.133; log-rank *P* = 0.008).

## 4 Discussion

In this retrospective study utilizing IVUS to evaluate patients undergoing PCI for CTO of the LAD, MB was identified in 23.8% of cases. Compared to patients without MB, those with MB demonstrated unique anatomical and imaging features, including shorter true CTO segment lengths, reduced calcification within the occluded region, and a higher prevalence of lesions located proximally. Despite these ostensibly more favorable lesion morphologies, the presence of MB was significantly correlated with worse long-term clinical outcomes. During a median follow-up period of two years, the MB group experienced a significantly elevated rate of MACE and clinically driven TLR relative to those without MB. Multivariate Cox proportional hazards analysis further substantiated MB as an independent predictor of 2-year MACE (hazard ratio: 2.173; 95% confidence interval: 1.031–4.667; *P* = 0.021), even after controlling for traditional cardiovascular risk factors. These results indicate that MB not only modifies lesion morphology in the setting of LAD CTO but also carries independent prognostic significance, with a potentially deleterious impact on both procedural success and clinical prognosis.

### 4.1 Mechanistic insights into the preferential localization of CTO proximal to myocardial bridge

While MB has traditionally been regarded as a benign anatomical variation, accumulating evidence suggests it plays a pivotal role in modifying local coronary hemodynamics. This alteration predisposes the segment proximal to the bridge to the development of atherosclerosis and CTO, rather than the tunneled or distal segments of the artery.

#### 4.1.1 Pathophysiological mechanisms linking MB to CTO formation

CTO development in the context of MB does not typically arise from disease within the bridged segment itself. Instead, the pre-bridge (proximal) portion of the artery is affected, largely due to hemodynamic disruptions instigated by MB. During systole, the mural coronary artery (MCA) undergoes dynamic compression by the MB, leading to retrograde flow, decelerated blood velocity, and disruption of laminar shear patterns upstream of the bridge (17). These hemodynamic perturbations result in regions of low or oscillatory wall shear stress (WSS), conditions well known to impair endothelial homeostasis and facilitate atherosclerotic development (18). In areas of diminished shear, endothelial nitric oxide synthase (eNOS) activity is suppressed, reducing the bioavailability of nitric oxide (NO), a critical molecule for maintaining vascular tone and endothelial function. The resultant deficiency in NO promotes vascular inflammation, platelet adhesion, and smooth muscle cell (SMC) proliferation (18). Simultaneously, enhanced generation of reactive oxygen species (ROS) contributes to oxidative stress, facilitating lipid accumulation, foam cell transformation, and extracellular matrix breakdown—hallmark processes in chronic occlusive disease (19).

#### 4.1.2 Why CTO localizes proximally, rather than within or distal to MB

In contrast to the hemodynamic profile of the proximal segment, the bridged and distal portions of the artery are subjected to elevated shear stress levels. These conditions have atheroprotective effects, including enhanced NO production, reduced platelet activation, and suppression of pro-inflammatory signaling pathways (1, 20). Both histological examinations and advanced imaging modalities have consistently shown that atherosclerotic lesions are seldom observed within the MB segment. Instead, plaques predominantly form just proximal to the MB and often exhibit eccentric morphologies (1).

Furthermore, the mechanical forces exerted by MB during repetitive systolic compression may induce chronic endothelial injury in the proximal segment. This mechanical trauma is associated with the release of potent vasoactive mediators such as endothelin-1 (ET-1) and activation of the angiotensin-converting enzyme (ACE) system (21, 22). Such alterations foster a local vascular milieu that is increasingly pro-thrombotic and pro-inflammatory, predisposing the segment to plaque destabilization and thrombotic occlusion, thereby culminating in CTO formation.

Collectively, MB serves not as the site of direct pathology but as a biomechanical trigger that generates a vulnerable upstream vascular environment conducive to atherogenesis and total occlusion. These mechanistic insights offer a plausible and evidence-supported rationale for the anatomical predilection of CTO lesions proximal to MB, as consistently observed in both our current analysis and prior research findings.

### 4.2 Reduced coronary calcification in MB-associated CTO lesions

In our analysis, IVUS demonstrated that patients with MB-related LAD CTO lesions exhibited a lower prevalence of calcified plaques and a reduced extent of calcification within the occluded segment compared to those without MB. This observation underscores a key structural divergence between the two patient cohorts and may reflect the distinct hemodynamic conditions imposed by the presence of MB. The repetitive systolic compression characteristic of MB alters local vascular wall mechanics and generates unique shear stress distributions that are less favorable to chronic arterial remodeling and calcific plaque development.

Specifically, the bridged region and its upstream segment—particularly the pre-bridge area—are subjected to disturbed flow patterns and diminished shear stress, a combination that is more commonly associated with non-calcified or mixed plaque morphologies (18, 23). Unlike calcified lesions, non-calcified plaques—often termed soft or vulnerable plaques—are defined by a large lipid-rich core, a thin fibrous cap, and an abundance of inflammatory cell infiltration, characteristics that render them susceptible to rupture (24). Although coronary artery calcification is traditionally considered a surrogate marker of chronic plaque stability and burden, its relative paucity in MB-associated CTO lesions does not equate to clinical quiescence. On the contrary, the predominance of non-calcified, unstable plaque—particularly proximal to the MB where adverse hemodynamic influences are concentrated—may signal a higher risk profile. This insight urges caution in interpreting low calcific burden in such lesions; although it may suggest technical ease in lesion crossing or reduced stent length, the underlying plaque vulnerability increases the likelihood of peri-procedural complications during PCI. Therefore, meticulous imaging assessment and vigilant post-intervention surveillance are essential when managing these patients.

### 4.3 Shorter CTO and stent length in MB-associated lesions

In our investigation, both the IVUS-assessed length of CTO lesions and the total stent length were significantly reduced in patients with MB compared to those without. This finding likely reflects the distinct anatomical and pathophysiological profile associated with MB, particularly its capacity to shield the tunneled segment from atherosclerotic involvement, thereby localizing disease predominantly to the proximal LAD.

Multiple mechanisms have been proposed to explain this relative resistance to plaque accumulation within MB segments. Firstly, the bridged arterial portion is anatomically segregated from surrounding perivascular adipose tissue, which is a recognized contributor to atherogenesis via pro-inflammatory paracrine signaling (25). Secondly, advanced imaging modalities such as optical coherence tomography (OCT) have revealed an absence of adventitial vasa vasorum in MB regions (26), potentially limiting the transvascular infiltration of inflammatory stimuli. Moreover, the cyclical systolic compression exerted on the tunneled artery may promote enhanced lymphatic drainage, thereby facilitating the removal of lipids and inflammatory cytokines from the vessel wall (27). Lastly, elevated or physiologically favorable wall shear stress within the MB region has been implicated in the upregulation of atheroprotective genes and maintenance of endothelial function (20).

While these factors may account for the shorter lesion length and reduced stent requirement, they also pose procedural complexities. Stenting into MB segments is generally avoided due to the risks of inadequate expansion under systolic compression and the potential for mechanical complications such as stent fracture or malapposition. As a result, the selection of appropriate landing zones becomes more constrained, particularly in MB-associated CTOs characterized by focal disease (8). Thus, although MB appears to offer a form of anatomical protection against extended atherosclerotic development, this benefit is offset by technical challenges in PCI planning.

The observed reduction in lesion and stent lengths in the MB cohort should not be viewed purely as a procedural advantage. Rather, it underscores a set of anatomical limitations that necessitate careful pre-procedural imaging, strategic lesion preparation, and individualized stenting approaches to minimize the risk of incomplete lesion coverage or geographic miss.

### 4.4 Clinical implications and revascularization strategy for MB-associated LAD CTO lesions

Our study demonstrates that the presence of MB in LAD CTO lesions is significantly associated with higher incidences of MACE (19.7% vs. 8.7%, *P* = 0.033) and TLR (18.0% vs. 6.7%, *P* = 0.016) at the 2-year mark. Moreover, MB was identified as an independent prognostic factor for MACE in multivariate analysis (HR = 2.173, *P* = 0.021). These results underscore the clinical importance of tailoring PCI strategies for this distinct anatomical and hemodynamic subgroup.

While earlier studies have reported favorable long-term survival in patients with isolated MB—approximately 98% over 11 years (28)—the outcomes are markedly less favorable when MB coexists with coronary artery disease (CAD), particularly in individuals undergoing PCI. Our findings align with growing evidence that stent implantation within MB segments may be associated with adverse outcomes, including increased neointimal hyperplasia, elevated rates of in-stent restenosis, and higher incidence of TLR (10, 29). Tsujita et al. observed a significant rise in TLR (from 3% to 24%) when stent deployment extended into MB regions (10). Additional studies using OCT and IVUS have revealed substantial neointimal tissue proliferation and even mechanical complications, such as stent fracture, within bridged segments (1).

These unfavorable outcomes are likely a result of the unique biomechanical environment inherent to MB. Stents positioned within MB segments are subjected to repetitive systolic compression, non-physiological shear stress patterns, and the so-called “sandwich effect”, in which the device is compressed between the coronary artery wall and the overlying myocardium (1, 30). This dynamic stress environment promotes the release of vasoactive substances, activates local inflammatory pathways, and may accelerate mechanical fatigue of the stent, thereby contributing to maladaptive remodeling and subsequent clinical complications.

From a technical perspective, the reduced CTO lesion length and the clinical preference to avoid stenting within MB segments result in limited options for stent landing zones. This constraint may elevate the risk of incomplete lesion coverage or geographic miss. Therefore, the presence of MB should be carefully evaluated during pre-procedural planning—ideally with high-resolution imaging modalities such as IVUS or CT—to facilitate accurate landing zone selection and optimal stent deployment strategy. Collectively, these findings suggest that conventional PCI algorithms may be insufficient for managing MB-associated LAD CTO lesions. Instead, a customized revascularization approach should be considered—one that aims to mitigate mechanical strain on the stent, avoid unnecessary implantation within bridged segments, and explore alternative therapies such as drug-coated balloon (DCB) angioplasty or ultrashort stent platforms. Additionally, rigorous post-PCI monitoring and extended follow-up are warranted to identify and manage potential TLR events or stent-related complications.

### 4.5 Limitations

This study has several noteworthy limitations that should be acknowledged. First, it was conducted as a single-center, retrospective observational study, which inherently introduces potential selection bias and limits the generalizability of the findings to other centers, populations, and practice settings. Second, various procedural decisions—including selection of stents, lesion preparation techniques, and strategy choice (antegrade vs. retrograde)—were made at the discretion of individual operators. This operator-dependent variability may have introduced heterogeneity that influenced clinical outcomes. Third, the analysis was confined to CTO lesions of the LAD, limiting the extrapolation of the results to CTO lesions in other coronary vessels such as the RCA or LCX, where the presence of MB is uncommon but may still be clinically relevant. Fourth, referral bias may have influenced the composition of the study population, complicating efforts to determine the true prevalence of MB in either LAD CTO or non-CTO scenarios. Fifth, no routine angiographic or intravascular imaging follow-up was performed after the index procedure, limiting our ability to evaluate stent-related complications such as in-stent restenosis, stent fracture, or neoatherosclerosis, and to correlate imaging findings with clinical outcomes. Sixth, although multivariate analysis was feasible for MACE, the relatively small number of events related to both MACE and TLR constrained the statistical power of the Cox regression models, resulting in wide confidence intervals and necessitating cautious interpretation of these findings. Seventh, subgroup analyses comparing clinical outcomes between patients with stenting within MB segments vs. those without, and interaction analyses incorporating clinical modifiers such as CTO score or lesion calcification, could not be performed due to limited sample size and low event rates in these subgroups. Lastly, although critical anatomical variables such as plaque burden and stent length are known to impact long-term clinical outcomes, these were not consistently matched or adjusted in subgroup comparisons. Despite these limitations, our findings are consistent with prior literature suggesting that stent placement within MB segments may predispose patients to unfavorable outcomes, including neointimal proliferation and restenosis (29, 31). Future multicenter, prospective studies with larger sample sizes are warranted to validate and extend these findings to broader patient populations.

## 5 Conclusion

In this IVUS-guided observational analysis of patients undergoing PCI for LAD CTO lesions, MB was associated with distinct anatomical characteristics, including shorter occlusion length, reduced calcific burden, and a higher prevalence of proximally located lesions. Although these morphological features may initially appear favorable, the presence of MB was independently linked to increased rates of MACE and TLR at two years. These results underscore the dualistic role of MB—as a structural feature that may shield against atherosclerosis within the bridged segment, yet simultaneously introduce procedural complexity and heightened clinical risk. Given the biomechanical constraints posed by MB, particularly in the setting of stent implantation, intervention within MB segments warrants a cautious and individualized approach. The risk of adverse outcomes such as stent malapposition, fracture, and restenosis necessitates careful pre-procedural planning and intravascular imaging to guide optimal landing zone selection and stent deployment. Overall, our findings emphasize the importance of treating MB-associated LAD CTO lesions as a distinct clinical entity, rather than applying conventional revascularization algorithms. Future investigations should aim to further elucidate the prognostic implications of MB in CTO interventions and to develop tailored treatment strategies that mitigate procedural risks while ensuring durable and effective revascularization.

## Data Availability

The original contributions presented in the study are included in the article/Supplementary Material, further inquiries can be directed to the corresponding author.
